# Seed germination in a southern Australian temperate seagrass

**DOI:** 10.7717/peerj.3114

**Published:** 2017-03-23

**Authors:** Erin Cumming, Jessie C. Jarvis, Craig D.H. Sherman, Paul H. York, Timothy M. Smith

**Affiliations:** 1 Centre for Integrative Ecology, School of Life and Environmental Sciences, Deakin University, Waurn Ponds, Victoria, Australia; 2 Department of Biology and Marine Biology, Center for Marine Science, University of North Carolina Wilmington, Wilmington, NC, United States; 3 Centre for Tropical Water & Aquatic Ecosystem Research, James Cook University, Cairns, Queensland, Australia

**Keywords:** Seagrass, Sediment, Temperature, Burial depth, Salinity, *Heterozostera tasmanica*, *Zostera nigricaulis*, Resilience

## Abstract

In a series of experiments, seeds from a temperate seagrass species, *Zostera nigricaulis* collected in Port Phillip Bay, Victoria, Australia were exposed to a range of salinities (20 PSU pulse/no pulse, 25 PSU, 30 PSU, 35 PSU), temperatures (13 °C, 17 °C, 22 °C), burial depths (0 cm, 1 cm, 2 cm) and site specific sediment characteristics (fine, medium, coarse) to quantify their impacts on germination rate and maximum overall germination. In southern Australia the seagrass *Z. nigricaulis* is a common subtidal species; however, little is known about the factors that affect seed germination which is a potential limiting factor in meadow resilience to natural and anthropogenic disturbances. Overall seed germination was low (<20%) with germination decreasing to <10% when seeds were placed in the sediment. When germination of *Z. nigricaulis* seeds was observed, it was enhanced (greater overall germination and shorter time to germination) when seeds were exposed to a 20 PSU pulse for 24 h, maintained at salinity of 25 PSU, temperatures <13 °C, in sediments with fine or medium grain sand and buried at a depth of <1 cm. These results indicate that germination of *Z. nigricaulis* seeds under *in situ* conditions may be seasonally limited by temperatures in southern Australia. Seed germination may be further restricted by salinity as freshwater pulses reaching 20 PSU are typically only observed in Port Phillip Bay following large scale rainfall events. As a result, these populations may be particularly susceptible to disturbance with only a seasonally limited capacity for recovery.

## Introduction

Seagrasses are a group of marine angiosperms that are a conspicuous element of coastal environments where they stabilize sediments (Orth et al., 2006; Ward, Michael Kemp & Boynton, 1984), provide food and habitat for economically important recreational and commercial fisheries species (Hemminga & Duarte, 2000), cycle nutrients (Den Hartog, 1970; Hemminga & Duarte, 2000; Orth et al., 2006), promote biodiversity (Beck et al., 2001; Orth et al., 2006; Short & Wyllie-Echeverria, 1996) and provide long term storage of organic carbon (Fourqurean et al., 2012; Orth et al., 2006). *Zostera nigricaulis* (Kuo) Jacobs and Les. (formerly referred to as *Heterozostera tasmanica*) is a common subtidal seagrass species found in southern Australia (Carruthers et al., 2007; Smith et al., 2013). In Port Phillip Bay *Z. nigricaulis* provides a major subtidal habitat to many species, however, the species has shown significant decline over the past decade that has been attributed to environmental conditions (Ball, Soto-Berelov & Young, 2014; Hirst et al., 2016). Globally, anthropogenic and natural impacts such as coastal development and storms (Orth et al., 2006) have led to a 29% global loss of seagrass over the last century (Waycott et al., 2009). Following these declines, it has become increasingly important to understand what factors are influencing seagrass resilience to stressors as well as limiting their recovery from disturbances.

Seagrasses reproduce both sexually via flowering and asexually through rhizome extension and vegetative fragmentation (Den Hartog, 1970; McMahon et al., 2014; Thomson et al., 2015). The resilience of seagrass ecosystems to disturbance relies on their ability to resist environmental stressors and to recover from loss via sexual and/or asexual reproductive mechanisms (Macreadie, York & Sherman, 2014; Orth et al., 2006; Unsworth et al., 2015). While seagrasses have the capability to recover from disturbance through the use of rhizomes (Frederiksen et al., 2004; Neckles et al., 2005; Rasheed, 1999), when complete above-ground biomass has been lost, initial re-establishment is determined by sexual reproduction in relation to seed germination and seed bank density (Greve et al., 2005; Plus, Deslous-Paoli & Dagault, 2003). Under these conditions seed bank germination rates are important factors influencing primary natural re-establishment of sexual recruits (Jarvis & Moore, 2010; Lee et al., 2007).

Seed germination is a potential limiting stage in successful sexual reproduction for both terrestrial (Harper, 1977) and marine angiosperms (Jarvis & Moore, 2015; Marion & Orth, 2012; Orth et al., 2000). Germination failure has predominantly been related to the characteristics of the surrounding microenvironment which may lack the required signals to break seed dormancy, which can last up to 12 months for other *Zostera* species (Orth et al., 2000), and initiate germination (Baskin & Baskin, 2014). Therefore, understanding those environmental cues that reduce time to germination and increase the maximum number of germinated seeds is essential to determine the potential for seagrass recovery via sexual reproduction. Germination cues have recently been identified as an important knowledge gap in Australian seagrass research (York et al., 2016).

Seagrass germination experiments have primarily focused on two species, *Zostera marina* (Abe, Kurashima & Maegawa, 2008; Jarvis & Moore, 2015; Marion & Orth, 2009; Moore, Orth & Nowak, 1993; Probert & Brenchley, 1999) and *Z. muelleri* (Brenchley & Probert, 1998; Conacher et al., 1994; Stafford-Bell, Chariton & Robinson, 2016). For both *Zostera* species temperature, salinity, anoxic conditions and burial depth have been documented to significantly affect germination success (Abe, Kurashima & Maegawa, 2008; Conacher, Poiner & O’Donohue, 1994; Jarvis & Moore, 2015; Probert & Brenchley, 1999). In general greater germination (defined as higher maximum germination and shorter time to germination) for both species occurs at lower temperatures (5 °C–16 °C) and salinities <20 PSU and under anoxic conditions (Brenchley & Probert, 1998; Conacher et al., 1994; Moore, Orth & Nowak, 1993; Orth & Moore, 1983; Probert & Brenchley, 1999; Van Lent & Verschuure, 1995). Of the few studies that investigated the effects of sediment and burial depth on *Zostera* seed germination, seeds buried between 1 cm and 5 cm had greater maximum germination and shorter time to germination than seeds found at depths greater than 5 cm (Wang et al., 2016) and that time to germination was also shorter in anoxic (Jarvis & Moore, 2010) or fine grained sediments compared to oxygenated coarse sediments (Wang et al., 2016). To date, environmental cues for *Z. nigricaulis* seed germination are undefined.

Recent studies have described *Z. nigricaulis* life history and the role seeds play in meadow maintenance (Smith et al., 2016a; Smith et al., 2016b; Thomson et al., 2015). The aim of this study was to add to this knowledge base by determining optimal germination conditions for *Z. nigricaulis* under controlled laboratory conditions by quantifying time to germination and maximum germination of seeds across a range of temperature, salinity, burial depths and sediment conditions.

## Methods

### Seed collection and storage

*Zostera nigricaulis* flowering shoots with mature spadices were collected in December 2012, during the period of maximum seed production (Smith et al., 2016b), from Blairgowrie (38°21′47″S, 144°47′28″E) in Port Phillip Bay, Victoria, Australia (Fig. S1). Samples were stored in flow through outdoor mesocosms (60 L volume, 60 × 35 × 30 cm) at the Victorian Marine Science Consortium (VMSC) in Queenscliff, Victoria under ambient conditions until seeds dehisced from the reproductive shoots. Seagrass samples were then sorted by hand and sieved (710 µm mesh) to separate mature seeds from remaining vegetative material (Marion & Orth, 2010). After separation, seeds were stored indoors in 1 L tanks with flow through seawater (∼21 °C) and light aeration (Jarvis & Moore, 2015; Jarvis & Moore, 2010). All seeds used in experiments were scarified using a scalpel under a dissecting microscope to create a slight opening in the seed coat to stimulate germination (Karrfalt, 2008). Any seeds that appeared damaged after scarification (e.g., embryo visible through the seed coat) or to have developed fungal growth were discarded. Previous research has shown few seeds with intact seed coats germinate (Conacher et al., 1994), therefore we chose to scarify seeds to promote seed germination and allow the effect of different environmental conditions on germination to be tested.

### Sediment collection and characterization

Three replicate sediment cores (2 cm width × 3 cm height) were collected from three sites (*n* = 9) which represented a range of observed different sediment types within *Z. nigricaulis* meadows (Blairgowrie) Avalon (38°05′08″S, 144°25′43″E) and Williamstown (37°52′14″S, 144°54′32″E) and stored in a cool room (4 °C) until processing. All sediment samples were analysed for percent organic matter and sediment grain size was quantified using standard methods (Erftemeijer & Koch, 2001). Organic matter was measured by drying sediment sample cores in a drying oven at 60 °C for 24 h followed by 5 h in a blast furnace (500 °C), with percentage of organic matter lost on ignition recorded after being weighed. To quantify grain size, sediment samples were exposed to hydrogen peroxide (30%) for 24 h to eliminate organic matter, weighed and sieved (>2 mm, 1–2 mm, 500 µm–1 mm, 250–500 µm, 125–250 µm, 62–125 µm, <62 µm fractions) and the weight of each amount of sediment left in each sieve was recorded (Erftemeijer & Koch, 2001).

### Temperature and salinity experiment

Maximum germination and time to germination of *Z. nigricaulis* seeds were assessed across a 3-way fully orthogonal design with treatments of temperature (3 levels: 13 °C, 17 °C, 22 °C), salinity (3 levels; 25 PSU, 30 PSU, 35 PSU) and low salinity pulse (2 levels: 24 h pulse in 20 PSU seawater and no pulse). Temperature and salinity concentrations were chosen to reflect the natural variation found in Port Phillip Bay, while the low salinity pulse was chosen to represent stressful environmental conditions (Lee et al., 2012; Walker, 1999). Fifty seeds were randomly allocated to one of four replicate petri dishes for each of the 18 treatments containing damp filter paper and 3–5 ml of saline solution and placed into a temperature control room with 12 h light cycles. Salinity of treatments was monitored daily and saline solution was added when necessary (every 2nd or 3rd day). Seeds were scored as successfully germinated when the cotyledon was extended 0.5 mm or more from the seed (Conacher et al., 1994; Jarvis & Moore, 2010). The number of germinated seeds and the salinity of each treatment was recorded weekly at the beginning of the experiment and then fortnightly until completion of the experiment 107 days later.

### Burial depth and sediment composition experiment

Following the completion of the salinity and temperature experiment, maximum germination of *Z. nigricaulis* seeds in sediment was assessed across a 2-way fully orthogonal design with treatments of burial depth (3 levels: 0 cm, 1 cm, 2 cm) and varying sediment composition based on grain size distribution (3 levels: fine (>25 % fine sediment), medium (10% fine sediment), and coarse (<5% fine sediment)). Sediment from each site was then divided into twelve 11 × 6 cm plastic experimental cores and 25 seeds were buried at the allocated depths. Based on the results of the previous experiment, all seeds were exposed to a fresh water pulse (20% PSU for 48 h) before burial to maximize germination. All cores were then randomly placed into 2 tanks (100 × 40 cm) with flow-through seawater (∼35 PSU). Tanks were held in a temperature control room (13 °C) with a 12 h light cycle for 7 weeks. Germination (cotyloid growth of 0.5 mm) was recorded every two weeks (Conacher et al., 1994; Jarvis & Moore, 2010) until the completion of the experiment after 50 days.

### Statistical analysis

Prior to the beginning of the experiments, a set of analytical models were developed to describe the relationship between maximum germination and mean time to germination (MTG) for each experiment. To determine the best fitting model, the Akaike’s Information Criterion corrected for small sample sizes (AICc) was calculated using loglikelihood ratios derived from all regression analyses (Burnham & Anderson, 2002). AICc differences between all models were then calculated and the models were ranked (Barton, 2013; R Core Team, 2014). The best-fitting model was considered to be the simplest model that fell within two of the lowest AICc (Burnham & Anderson, 2002; Tables S1–S5). Overall effects of categorical variables on MTG and maximum germination were calculated with Wald Chi square tests using the ‘lmtest’ package (Zeileis & Hothorn, 2002).

Based on the large numbers of zeros found within the treatments (∼43%) and overall low germination response in both experiments, the effects of experimental treatments on maximum germination of *Z. nigricaulis* seeds were analysed using a zero/one inflated beta binomial (ZOIB) regression with the ‘gamlss’ package (Rigby & Stasinopoulos, 2005) in the statistical program R (R Core Team, 2014). ZOIB regression models can be used to model response variables that are bound between or equal to 0 or 1 and contain a non-negligible number of zeros and or ones (Ospina & Ferrari, 2010).

Due to the potential for a large amount of right-censored data characteristic of germination experiments (McNair, Sunkara & Frobish, 2012; Scott, Jones & Williams, 1984), Cox models were selected to quantify treatment effects (pulse and non-pulsed seeds, temperature and salinity, burial depth and sediment source) on mean time to germination (MTG). Seed data were censored if seed germination did not occur and non-germinated seeds were flagged prior to analysis. As time-event analyses are based on the distribution of germination times of individual seeds rather than on cumulative germination curves, each seed was analysed independently using the survival package (Therneau & Grambsch, 2000). Germination data were first graphically explored for violations of the proportional hazards function by plotting separate non-parametric Kaplan–Meier survivorship functions for the different factors (McNair, Sunkara & Frobish, 2012), KM surv (Klein & Moeschberger, 2005). Potential multicollinearity of the covariates were tested by calculating variance inflation factors (Heiberger & Holland, 2015) for all treatment factors prior to analysis. The effects of treatment on time to germination were then calculated using the Cox model (survival, Therneau & Grambsch, 2000). Seed germination was only recorded at the end of the sediment experiment and therefore MTG was not calculated.

Sediment grain size and percentage organic matter data were calculated as proportions of total sample weight and independently analysed using SYSTAT 12 with a one-way analysis of variance to compare between treatments (Quinn & Keough, 2002). Prior to analysis all sediment data were transformed when necessary to meet the assumptions of normality and homogeneity of variance (Quinn & Keough, 2002). All post hoc analyses of the data were performed with Tukey’s test.

## Results

### Salinity and temperature experiment

**Figure 1:**
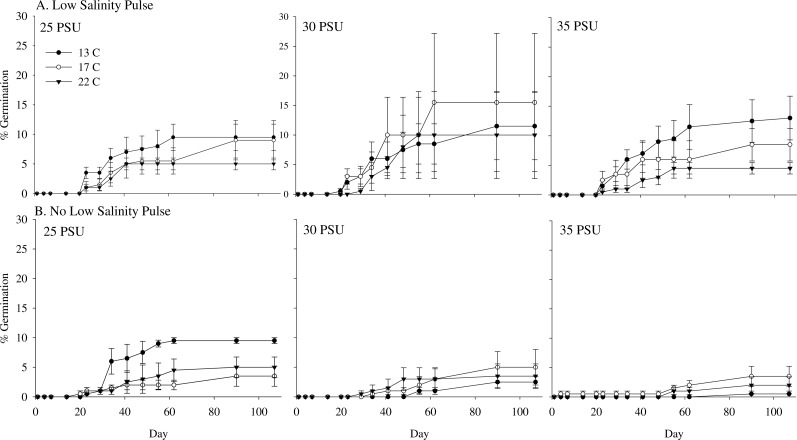
Maximum *Z. nigricaulis* percent germination in salinity experiment. Comparison of maximum percent germination (±mean SE) of *Z. nigricaulis* over 107 days with exposure to a low salinity pulse, three salinities (25 PSU, 30 PSU and 35 PSU) and three temperatures (13, 17, 22 °C).

Overall, maximum germination across all treatments was low (<20%; Table 1), however, seeds were more likely to germinate when exposed to a low salinity pulse (*p* < 0.001; Table 2). There was a significant interaction between pulse and salinity treatments (Wald chi square test, *p* = 0.047) with germination decreasing with increasing salinity in the non-pulsed treatment and no effect of salinity on germination in the seeds exposed to a low salinity pulse (Fig. 1 and Table 2). The inclusion of temperature in the model did not improve model fit and therefore was removed from the maximum germination analysis (Table S1).

**Table 1:** Maximum germination (Max % G) and mean time to germination (MTG) across all treatments in the salinity and temperature experiment (±SE).

**Pulse**	**Temp (°C)**	**Salinity**	**MTG (days)**	**Max % G**
Yes	25	13	37 ± 1	10 ± 2
		17	56 ± 1	9 ± 3
		22	50 ± 1	5 ± 1
	30	13	51 ± 1	12 ± 6
		17	54 ± 2	16 ± 12
		22	44 ± 1	10 ± 7
	35	13	54 ± 1	14 ± 4
		17	48 ± 1	9 ± 3
		22	42 ± 1	5 ± 1
No	25	13	42 ± 1	10 ± 1
		17	55 ± 2	4 ± 2
		22	47 ± 1	5 ± 2
	30	13	76 ± 3	3 ± 1
		17	71 ± 2	6 ± 3
		22	48 ± 1	4 ± 2
	35	13	43 ± 2	1 ± 1
		17	71 ± 2	4 ± 2
		22	73 ± 3	3 ± 1

**Table 2:** Zero/one inflated beta regression results for maximum germination of *Z. nigricaulis* seeds across pulse, salinity and temperature treatments.

Parameter	Coef.	SE	*t* value	*p*-value
Intercept	−3.542	0.289	−12.256	<0.001^*^
Pulse	1.300	0.343	3.793	<0.001^*^
Salinity 25 PSU	–	–	–	–
Salinity 30 PSU	0.633	0.346	1.832	0.072
Salinity 35 PSU	0.881	0.354	2.488	0.016^*^
Pulse:Sal (25 PSU)	–	–	–	–
Pulse:Sal (30 PSU)	−0.284	0.467	0.608	0.545
Pulse:Sal (35 PSU)	−0.930	0.459	−2.026	0.047^*^

**Notes.**

* indicates a significant value.

**Figure 2:**
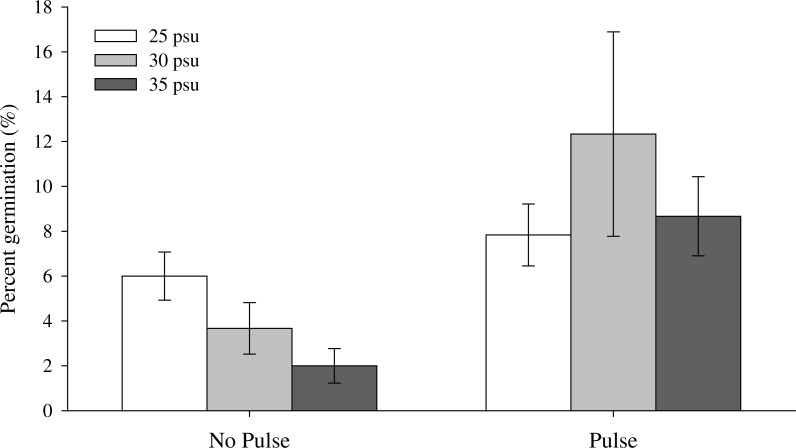
Maximum germination of *Z. nigricaulis* seeds for the salinity and temperature experiment. White bars indicate the 25 PSU treatment, light grey is 30 PSU and the dark grey represent the 35 PSU treatment.

Mean time to germination ranged from 37 ± 1 to 76 ± 1 days across all treatments. There was a significant interaction on mean time to germination between salinity, temperature and low salinity pulse treatments (*p* = 0.023, Fig. 2 and Table 3). Mean time to germination was on average 10 days earlier in the pulsed treatment (48 ± 6 days) compared to the non-pulsed treatment (58 ± 14 days) and in low salinity (48 ± 17 days) compared to medium (57 ± 13 days) and high salinity treatments (55 ± 14 days) (Table 1). Mean time to germination was shorter in the 13 °C (51 ± 14 days) and 22 °C (51 ± 11 days) treatments compared to 17 °C (59 ± 10 days) (Table 1). The shortest MTG (37 ± 1 day) occurred with seeds exposed to a low salinity pulse and exposed to salinities of 25 PSU and temperatures of 13 °C (Table 1).

**Table 3:** Results of survival analysis looking at differences in the main effects for pulse, salinity and temperature on mean time to germination of *Z. nigricaulis* seeds.

**Parameter**	**Coef.**	**Exp**	**SE**	***Z* score**	***p*-value**
Pulse	−12.540	0.000	5.033	−2.492	0.013^*^
Salinity	−0.402	0.669	0.150	−2.691	0.007^*^
Temperature	−0.518	0.596	0.240	−2.162	0.031^*^
Pulse: Salinity	0.493	1.637	0.174	2.840	0.005^*^
Pulse: Temp	0.596	1.816	0.287	2.080	0.038^*^
Salinity: Temp	0.018	1.018	0.008	2.119	0.034^*^
Pulse: Salinity: Temp	−0.022	0.978	0.010	−2.277	0.023^*^

**Notes.**

* indicates a significant value.

### Sediment type and burial depth experiment

**Figure 3:**
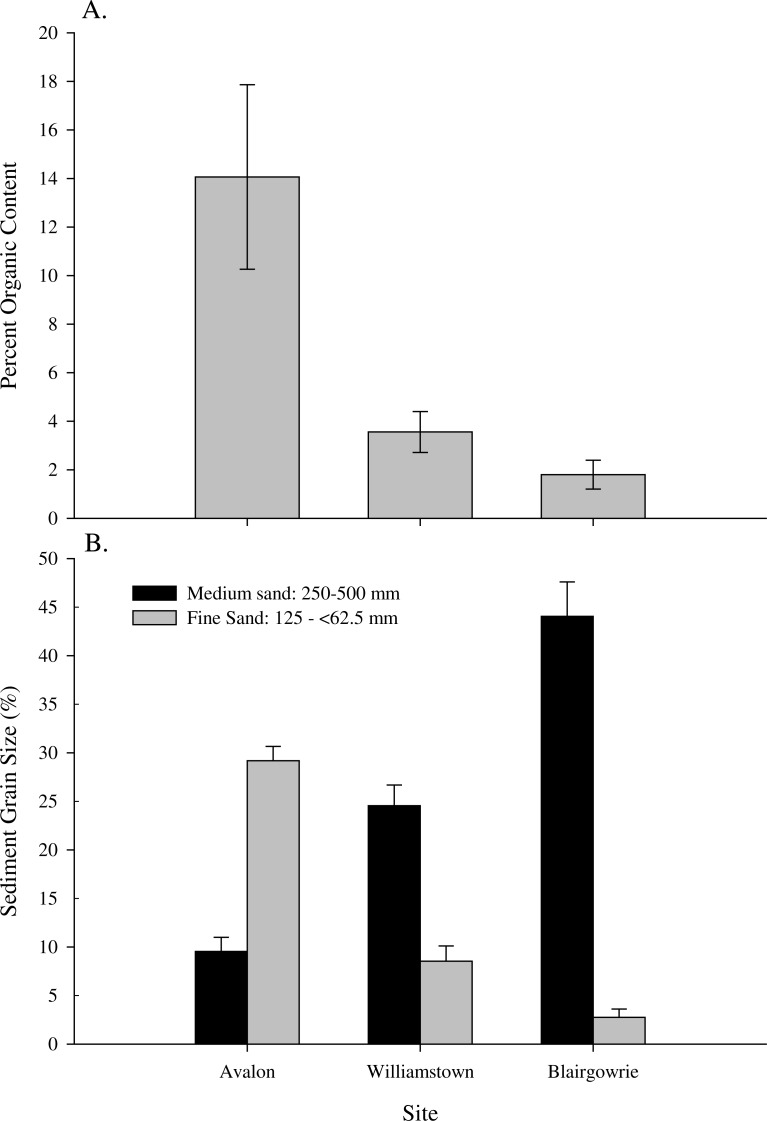
Experimental sediment characterization. (A) Mean (±SE) % organic matter content at Avalon, Williamstown and Blairgowrie and (B) mean (±SE) % of medium (250–500 µm) and fine (62–125 µm) grain sediment at Avalon, Williamstown and Blairgowrie.

Organic matter was significantly higher at Avalon than Williamstown and Blairgowrie (*F*_2,6_ = 8.51, *p* = 0.018, Fig. 3A). Of the seven sediment grain sizes measured only two showed any significant difference across sites. The proportion of medium grain sand (250–500 µm) was highest at Blairgowrie, followed by Avalon and Williamstown (*F*_2,6_ = 43.9, *p* < 0.001, Fig. 3B). Very fine grain sand (65–125 µm) was highest at Williamstown followed by Avalon and Blairgowrie which were not significantly different (*p* = 0.054). Therefore, based on organic matter content and grain size distribution, Williamstown sediment was used for the ‘fine’ sediment treatment, Avalon for the ‘medium’ and Blairgowrie for ‘coarse’ treatments.

**Figure 4:**
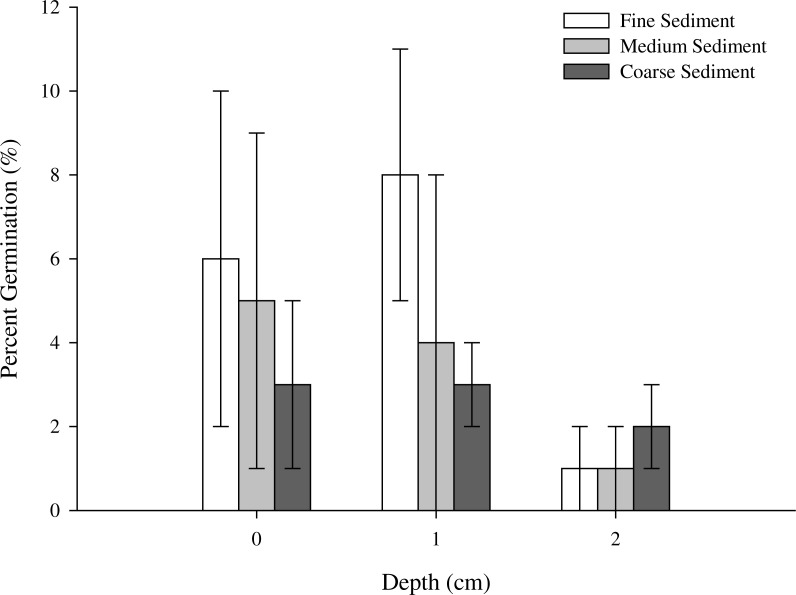
Maximum germination of *Z. nigricaulis* seeds (mean ± SE) for sediment type and burial depth experiment. The white bars represent fine, light grey represent medium and the dark grey bars represent coarse sediment grain size treatments.

When placed in sediment, maximum germination fell below 10% regardless of burial depth or sediment type (Table 4). Germination counts were not made frequently enough to calculate mean time to germination; however, maximum germination was significantly affected by the interaction between sediment type and burial depth (Wald Chi square test, *p* = 0.009). While there was no significant difference in germination between seeds buried at 0 and 1 cm (*p* = 0.660) or between seeds at 1 and 2 cm (*p* = 0.721), seeds placed on the sediment surface (0 cm) had a greater maximum germination than seeds buried at 2 cm (*p* = 0.048; Fig. 3; Table 5). Overall, seeds in Williamstown and Avalon sediment had greater germination than seeds placed in Blairgowrie sediment except at 2 cm depths (Fig. 4; Table 5).

**Table 4:** Maximum *Z. nigricaulis* germination (% G) across all treatments in the sediment type and burial depth experiment. Values are given as mean ± SE.

**Sediment type**	**Burial depth (cm)**	**Max % G**
Fine	0	6 ± 4
	1	8 ± 3
	2	1 ± 1
Medium	0	5 ± 4
	1	4 ± 4
	2	3 ± 1
Coarse	0	3 ± 2
	1	3 ± 1
	2	2 ± 1

**Table 5:** Zero/one inflated beta regression results for maximum germination of *Z. nigricaulis* seeds for sediment type and burial depth treatments.

**Parameter**	**Coef.**	**SE**	***t* value**	***p*-value**
Intercept	−2.013	0.214	−9.391	<0.001^*^
Fine	–	–	–	–
Medium	−0.232	0.2287	−0.810	0.426
Coarse	−0.719	0.355	−2.028	0.054
Depth 0 cm	−0.126	0.282	−0.446	0.660
Depth 1 cm	−0.126	0.282	−0.446	0.660
Depth 2 cm	−1.055	0.505	−2.088	0.048^*^
Fine: depth (1)	–	–	–	–
Medium: depth (1)	0.006	0.419	0.014	0.989
Coarse: depth (1)	−0.210	0.478	−0.439	0.665
Fine: depth (2)	–	–	–	–
Medium: depth (2)	1.667	0.602	2.769	0.011^*^
Coarse: depth (2)	0.719	0.663	1.085	0.289

**Notes.**

* indicates a significant value.

## Discussion

Surprisingly, overall germination of *Z. nigricaulis* seeds across all experiments was low (<20%) compared to other *Zostera* species. Germination rates of *Z. muelleri* (formerly *Z. capricorni*), a co-occurring intertidal species, range between 20%–60% under similar conditions (Brenchley & Probert, 1998; Conacher et al., 1994) while temperate northern hemisphere species *Z. marina* and *Z. noltii* range from 5%–100% and <10%–80% respectively (Hootsmans, Vermaat & Van Vierssen, 1987; Van Lent & Verschuure, 1995). However, high germination rates in many of these studies were at very low salinity levels (<20 PSU) that are rarely encountered in the field (Lee et al., 2012; Probert & Brenchley, 1999). When considering salinities of 25–40 PSU, which overlap with this experiment and are more reflective of natural conditions observed in Port Phillip Bay, *Z. muelleri* germination was <20% in all treatments except when temperatures were <16 °C (Brenchley & Probert, 1998; Conacher et al., 1994). While germination was also greatest at low temperatures in this study (25 PSU at 13 °C), the overall low germination response indicates that additional germination cues (e.g., dissolved oxygen, light, variations in sediment microbial communities) both individually or in combination may be missing and require further investigation.

Salinity and temperature are key germination cues for many *Zostera* species (Conacher et al., 1994; Hootsmans, Vermaat & Van Vierssen, 1987; Kaldy et al., 2015; Orth & Moore, 1983; Stafford-Bell, Chariton & Robinson, 2016; Van Lent & Verschuure, 1995). Seeds of *Z. nigricaulis* generally had greater and quicker germination in lower salinities and temperatures. Although consistent with other germination studies recorded for *Z. muelleri*, *Z. marina* and *Z. noltii* (Brenchley & Probert, 1998; Conacher et al., 1994; Probert & Brenchley, 1999; Van Lent & Verschuure, 1995), high germination at low salinities seems an unlikely germination cue for *Z. nigricaulis*. *Zostera nigricaulis* is found in large bays and protected coastal habitats but is absent from estuaries suggesting it has little tolerance of low salinities. Lower salinities promoted higher and faster germination rates and in most cases a low salinity pulse increased germination. In contrast, *Z. muelleri* and *Z. marina* are often found in estuaries and therefore greater germination at low salinities may have important ecological implications. Salinities in Port Phillip Bay rarely reach the level of 20–30 NTU that was used for a 24 h pulse and as a low salinity treatment and there are few freshwater inputs to reduce salinities under flood conditions (Lee et al., 2012; Walker, 1999). Flood conditions that lower salinities and cause disturbance creating space for seeds to grow into could explain high germination in low salinity; however, floods are generally associated with high sediment and turbidity levels that may bury seeds and restrict light for seedling growth.

Variation in levels of *Z. nigricaulis* seed germination at different temperatures and salinities may have important ecological implications. Temperature and salinity values used in this study were chosen to reflect the range within Port Phillip Bay. Peak flowering in *Z. nigricaulis* occurs during October and November each year (Smith et al., 2016b), which coincides with mean seawater temperatures between 13 °C and 17 °C. Once germinated, seedling growth may be rapid at this time of the year as temperatures and daylight increase moving into the Austral summer. Germinating at the onset of optimal growing conditions increases the length of the peak growing period for seedlings increasing their chance of survival. This reflects seed germinations in other species that coincides with high seed banks and proceeds the growing season (Garwood, 1983).

Changes in global climate conditions will impact plant species reproduction and resilience strategies. In Port Phillip Bay, water temperature and salinity are expected to increase over the next 15 years with salinity expected to increase by as much as 4 NTU in the Geelong Arm of Port Phillip Bay (Lee et al., 2012). Already the Geelong Arm has sustained considerable seagrass loss (Ball, Soto-Berelov & Young, 2014) and further increases in salinity will restrict the ability of seagrass to recover from seeds as germination decreases. Seeds are often an important recovery mechanism in seagrass ecosystems (Alexandre, Santos & Serrao, 2005; Hammerstrom et al., 2006; Jarvis & Moore, 2010) but changing conditions in the future will have major implications for the ability of seagrass to recover and reduce the resilience of seagrass habitats. To fully understand the impacts of environmental changes in near shore coastal environments, and to gain a better understanding of potential resilience of this species to increased disturbance, additional research is required to better understand seed ecology.

### Sediment conditions

In addition to salinity and temperature, germination of *Z. nigricaulis* seeds is affected by both burial depth and sediment type, consistent with previous studies in other *Zostera* species (Jarvis & Moore, 2015; Wang et al., 2016). Germination in fine and medium sediment was greater when seeds were at the sediment surface than when they were buried at 2 cm. Thus it is clear from this and previous studies that burial depth can affect *Zostera* germination (Granger, Traber & Nixon, 2000; Jarvis & Moore, 2015) and suggests that either germination cues interact with burial depth to affect germination rates, or that seeds germinate but do not have the energy stores required for the cotyledon to reach the surface (Jarvis & Moore, 2015; Wang et al., 2016). Seagrass beds accumulate sediment and organic matter (Bos et al., 2007; Fonseca & Fisher, 1986), burying seeds and consequently reducing germination rates. Therefore, although there may be a significant seed bank, as sediment is deposited over time, the likelihood of germination is lower and potentially decreases the ability of patches to recover from disturbances. Similarly, large scale along shore sediment movement can play a role in seagrass distributions and seagrass loss can occur through burial (Ball, Soto-Berelov & Young, 2014). These results suggest that in such a situation recovery is unlikely to occur from the seed bank.

Germination in coarse sediment was lower than in other grain sizes and showed no difference across depths. Variations in germination in different sediment types can be attributed to a variety of factors such as organic matter, grain size and anoxia (Jarvis & Moore, 2015; Tanner & Parham, 2010; Wang et al., 2016). High organic matter in sediment can lead to anoxic conditions, which can have higher germination rates than aerobic conditions (Brenchley & Probert, 1998; Probert & Brenchley, 1999). Organic matter in the coarse sediment was lower than at the other sites and therefore may be playing an important role in determining germinations rates. Likewise, sediment grain size is thought to affect sediment nutrient levels and can affect time to germination (Jarvis & Moore, 2015; Wang et al., 2016). Finer sediment in seagrass beds reduces pore water loss, increasing nutrient levels while in coarse sediment pore water, and nutrients are easily lost (Koch, 2001). Variations in nutrient levels in the different sediment treatments may explain differences in *Z. nigricaulis* seed germination but the role sediment nutrients levels play in seagrass seed germination is unknown (Jarvis & Moore, 2015). Further research into the impacts of varying sediment conditions are needed to explain why germination rates vary across sediment conditions.

Like many plant species seagrasses invest large amounts of energy into the production of seeds often producing vast quantities that enter the seed bank (e.g., Smith et al., 2016b). Seed banks are important for recovery and maintenance in many ecosystems that are susceptible to local habitat loss. Low germination rates may impact the resilience of *Z. nigricaulis* to disturbance and future environmental change. Inability to recover can have implications for coastal ecosystems given the many ecosystem services and therefore more research to better understand seagrass seed ecology.

##  Supplemental Information

10.7717/peerj.3114/supp-1 Table S1 A priori model selection for *Z. nigricaulis* seed germination in the salinity and temperature experiment *k* is the number of estimable parameters in the model.

10.7717/peerj.3114/supp-2 Table S2 Model selection results for mean time to germination of *Z. nigricaulis* seeds for the pulse, temperature and salinity experiment Model selection was based on calculated AICc values (Burnham & Anderson, 2002).

10.7717/peerj.3114/supp-3 Table S3 Model selection results for maximum germination of *Z. nigricaulis* seeds for the pulse, temperature and salinity experiment Model selection was based on calculated AICc values (Burnham & Anderson, 2002).

10.7717/peerj.3114/supp-4 Table S4 A priori model selection for *Z. nigricaulis* seed germination in the burial depth and sediment type experiment *k* is the number of estimable parameters in the model.

10.7717/peerj.3114/supp-5 Table S5 Model selection results for maximum germination of *Z. nigricaulis* seeds for the sediment type and burial depth experiment Model selection was based on calculated AICc values (Burnham & Anderson, 2002).

10.7717/peerj.3114/supp-6 Figure S1 Sampling sites in Port Phillip Bay, Victoria, Australia

10.7717/peerj.3114/supp-7 Supplemental Information 1 Statistical analysis methods for selection of analytical models

## Additional Information and Declarations

### Data Availability

The following information was supplied regarding data availability: Cumming, Erin and Smith, Timothy 2016, Zostera nigricaulis seed germination experiment [data collection], doi: 10.4225/16/5848b8f8d273b.
