# Supplementary material for: Seed germination in a southern Australian temperate seagrass
Source: PeerJ. 2017 Mar 23;5:e3114. doi: 10.7717/peerj.3114 (PMC5366064; doi:10.7717/peerj.3114)
Supplement: Table S1 — k is the number of estimable parameters in the model. [file peerj-05-3114-s001.docx]

| **Model** | **Intercept** | **Pulse** | **Salinity** | **Temperature** | **Pls:Sal** | **Pls:Tmp** | **Sal:Tmp** | **Pls:Sal:Tmp** | **k** |
| --- | --- | --- | --- | --- | --- | --- | --- | --- | --- |
| M1 | + |  |  |  |  |  |  |  | 1 |
| M2 | + | + |  |  |  |  |  |  | 2 |
| M3 | + |  | + |  |  |  |  |  | 2 |
| M4 | + |  |  | + |  |  |  |  | 2 |
| M5 | + | + | + |  |  |  |  |  | 3 |
| M6 | + | + |  | + |  |  |  |  | 3 |
| M7 | + |  | + | + |  |  |  |  | 3 |
| M8 | + | + | + | + |  |  |  |  | 4 |
| M9 | + | + | + |  | + |  |  |  | 4 |
| M10 | + | + |  | + |  | + |  |  | 4 |
| M11 | + |  | + | + |  |  | + |  | 4 |
| M12 | + | + | + | + | + |  |  |  | 5 |
| M13 | + | + | + | + |  | + |  |  | 5 |
| M14 | + | + | + | + |  |  | + |  | 5 |
| M15 | + | + | + | + | + | + |  |  | 6 |
| M16 | + | + | + | + | + |  | + |  | 6 |
| M17 | + | + | + | + |  | + | + |  | 6 |
| M18 | + | + | + | + | + | + |  | + | 7 |
| M19 | + | + | + | + | + |  | + | + | 7 |
| M20 | + | + | + | + |  | + | + | + | 7 |
| M21 | + | + | + | + | + | + | + | + | 8 |
