# Supplementary material for: Seed germination in a southern Australian temperate seagrass
Source: PeerJ. 2017 Mar 23;5:e3114. doi: 10.7717/peerj.3114 (PMC5366064; doi:10.7717/peerj.3114)
Supplement: Table S3 — Model selection was based on calculated AICc values (Burnham & Anderson, 2002). [file peerj-05-3114-s003.docx]

| **Model** | **Int.** | **Pulse** | **Salinity** | **Temp** | **Pls:Sal** | **Pls:Tmp** | **Sal:Tmp** | **Pls:Sal:Tmp** | **k** | **AICc** | **Delta** | **Rank** |
| --- | --- | --- | --- | --- | --- | --- | --- | --- | --- | --- | --- | --- |
| M9 | + | + | + |  | + |  |  |  | 4 | -269.40 | 0.00 | 1 |
| M12 | + | + | + | + | + |  |  |  | 5 | -268.69 | 0.71 | 2 |
| M5 | + | + | + |  |  |  |  |  | 3 | -268.18 | 1.22 | 3 |
| M15 |  | + | + | + | + | + |  |  | 6 | -265.36 | 3.77 | 4 |
