# Supplementary material for: Seed germination in a southern Australian temperate seagrass
Source: PeerJ. 2017 Mar 23;5:e3114. doi: 10.7717/peerj.3114 (PMC5366064; doi:10.7717/peerj.3114)
Supplement: Table S4 — k is the number of estimable parameters in the model. [file peerj-05-3114-s004.docx]

| **Model** | **Intercept** | **Sed** | **Depth** | **Sed:Depth** | **k** |
| --- | --- | --- | --- | --- | --- |
| B1 | + |  |  |  | 1 |
| B2 | + | + |  |  | 2 |
| B3 | + |  | + |  | 2 |
| B4 | + | + | + |  | 3 |
| B5 | + | + | + | + | 4 |
