# Supplementary material for: Seed germination in a southern Australian temperate seagrass
Source: PeerJ. 2017 Mar 23;5:e3114. doi: 10.7717/peerj.3114 (PMC5366064; doi:10.7717/peerj.3114)
Supplement: Table S5 — Model selection was based on calculated AICc values (Burnham & Anderson, 2002). [file peerj-05-3114-s005.docx]

| **Model** | **k** | **AIC** | **AICc** | **Delta** | **Rank** |
| --- | --- | --- | --- | --- | --- |
| B2 | 2 | -17.04 | -16.87 | 0.00 | 1 |
| B5 | 4 | -13.94 | -13.34 | 3.53 | 2 |
| B4 | 3 | -13.66 | -13.31 | 3.56 | 3 |
| B1 | 1 | -12.26 | -12.21 | 4.66 | 4 |
