# Supplementary figures and images for: Seed germination in a southern Australian temperate seagrass
Source: PeerJ. 2017 Mar 23;5:e3114. doi: 10.7717/peerj.3114 (PMC5366064; doi:10.7717/peerj.3114)

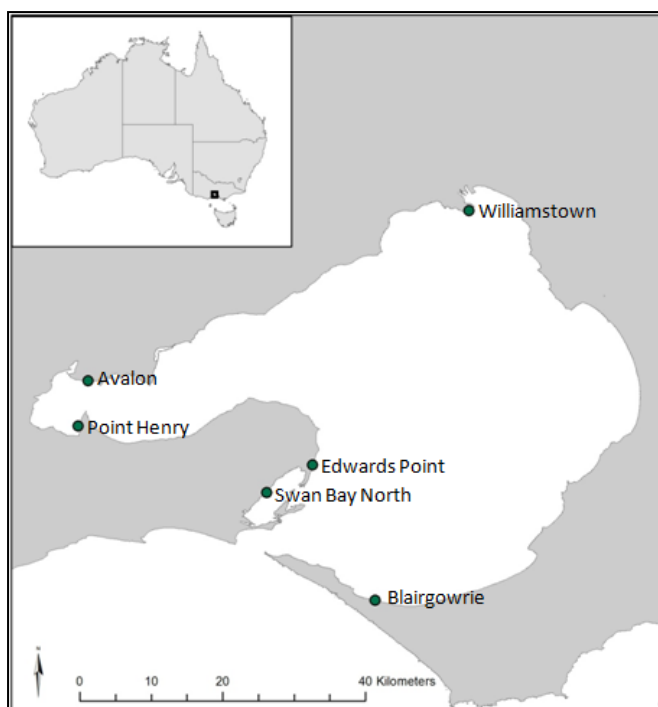

Supplement: Figure S1 [file peerj-05-3114-s006.pdf]
