# Supplementary material for: Seed germination in a southern Australian temperate seagrass
Source: PeerJ. 2017 Mar 23;5:e3114. doi: 10.7717/peerj.3114 (PMC5366064; doi:10.7717/peerj.3114)
Supplement: Supplemental Information 1 [file peerj-05-3114-s007.docx]

Statistical Analysis

Prior to the beginning of the experiments, a set of analytical models were developed to describe the relationship between maximum germination and Mean Time to Germination (MTG) for each experiment. To determine the best fitting model, the Akaike’s Information Criterion corrected for small sample sizes (AICc) was calculated using loglikelihood ratios derived from all regression analyses (Burnham and Anderson 2002). AICc differences between all models were then calculated and the models were ranked (MuMIn, Barton 2013; R Core Team 2014). The best-fitting model was considered to be the simplest model that fell within two of the lowest AICc (Burnham and Anderson 2002).
